# Infections in Patients with Rheumatoid Arthritis in the Era of Targeted Synthetic Therapies

**DOI:** 10.31138/mjr.31.1.129

**Published:** 2020-06-11

**Authors:** Konstantinos Thomas, Dimitrios Vassilopoulos

**Affiliations:** 1 4^th^ Department of Medicine, Attikon University Hospital, National and Kapodistrian University of Athens, School of Medicine, Athens, Greece; 2 Clinical Immunology-Rheumatology Unit, 2^nd^ Department of Medicine and Laboratory, Hippokration General Hospital, National and Kapodistrian University of Athens, School of Medicine, Athens, Greece

**Keywords:** Rheumatoid arthritis, infections, biologics, JAK inhibitors, tuberculosis, vaccinations, hepatitis B virus

## Abstract

The third decade of the 21^st^ century marks the beginning of a new era in the treatment of rheumatoid arthritis (RA). Recently, after the introduction in clinical practice of different biologics in the first decade, three different oral synthetic targeted agents (JAK inhibitors) have been licensed for the treatment of RA, in patients who had failed or are intolerant to disease modifying anti-rheumatic drugs (DMARDs). Despite the significant progress that these agents bring to the care of RA patients, the risk of infections is still present and clear, given that their risk for serious infections is at least comparable with that of biologic DMARDs, whereas the incidence of herpes zoster is higher than that of bDMARDs. Here, we review the most recent data regarding the risk for serious and opportunistic infections in RA patients treated with biologics or JAK inhibitors, as well the up-to-date approach for managing and preventing such infections in RA patients.

## INTRODUCTION

Rheumatoid arthritis (RA) is the most common inflammatory arthritis, with an estimated worldwide prevalence between 0.5–1%.^1^ Despite the tremendous improvement that biologic (b-) and targeted synthetic (ts-) disease-modifying anti-rheumatic drugs (DMARDs) brought to its management, RA patients still carry higher mortality compared to the general population; however, this gap seems to be gradually closing in the recent years.^2^

Infections are considered one of the most important comorbidities in patients with RA associated with excess morbidity and mortality. Here, we review the most common infections in patients with RA, the role of immunosuppressive therapies (with a special emphasis on ts-DMARDs), and the currently available preventive strategies.

## BACTERIAL INFECTIONS

Bacterial infections are by far the most common causes of serious infections in patients with RA.^3–6^ The sites most often affected are the lung, urinary tract, and skin/skin structures.^6,7^ There are no major differences in the type of isolated pathogens compared to the general population, possibly with the exception of intracellular bacteria in patients treated with tumour necrosis factor-α inhibitors (TNFi).

Data accumulated over the last 20 years from randomized controlled studies (RCTs) and their long-term extension studies, and real-life data from patient registries, have shown that the incidence of serious infections (ie, requiring hospitalization or IV antibiotics) in RA patients ranges significantly between 1.5–7/100 patient-years.^8^

A number of patient, disease and treatment characteristics have been identified as risk factors for the development of serious infections (*Table 1*). These include older age, history of serious infection, functional disability (high HAQ score), certain comorbidities (especially chronic lung or kidney disease), high daily dose of glucocorticoids (GCs, >7.5 mg/day) and previous treatment failures (with biologics or non-biologics).^6,8–10^ History of previous infections and high disease activity are probably the most important drivers of that risk in RA patients.^4 ,9–14^

**Table 1.** Risk factors associated with serious infections in patients with rheumatoid arthritis.

**Older age (> 65 years)**
**High disease activity (i.e. DAS28-score)**
**High disability score (i.e. HAQ score)**
**Comorbidities (i.e. chronic lung or kidney disease)**
**Glucocorticoid treatment (> 7.5 mg/day)**
**History of previous serious infections**
**Current immunosuppressive therapy (b- or ts-DMARDs)**
**History of previous DMARD failures**

DAS28: Disease Activity Score using 28 joints, HAQ: Health Assessment Questionnaire, b-DMARDs: biologic disease modifying anti-rheumatic drugs, ts-DMARDs: targeted synthetic DMARDs. See text for details and from references: ^6^, ^8–10^.

## THE ROLE OF ANTI-RHEUMATIC THERAPIES

### csDMARDs - Glucocorticoids

Regarding conventional synthetic-DMARDs (cs-DMARDs) such as methotrexate or leflunomide, there are reassuring data to suggest that they confer either a small or no increased risk for serious infections in RA patients.^3,9,10^ On the contrary, for GCs, a dose-dependent increase in the risk of serious infections has been shown.^3,10,15^ Although daily doses below 5–7.5 mg are frequently considered as “safe”,^16^ it appears that long-term, continuous exposure even to low GC doses may also contribute to increased risk.^3,9^

### Biologic DMARDs

A significant body of evidence has been mounted from RCTs and real-world data regarding the infection risk of the different classes of bDMARDs.^8^ Most of the available data show that the overall incidence of serious infections in bDMARD-treated RA patients ranges between 3–7/100 patient-years, without major differences among the different classes of biologics (anti-TNFs, anti-B cell -rituximab, anti-T/APC cell inhibitors – abatacept, anti-IL6 inhibitors).^5,7,8,17,18^ This risk appears to be higher during the first year after treatment initiation.^15,19^

After the first serious infection, increased vigilance is required for early signs of a new infection, given that these patients have a 3–5 fold increased risk for a subsequent episode.^10,12,14,15^ Interestingly, despite their higher risk for bacterial infections, patients who develop sepsis while on bDMARDs have a lower risk for death compared to patients who are on csDMARDs (OR=0.56).^20^

### JAK inhibitors

Janus kinase (JAK) inhibitors (baricitinib, tofacitinib, upadacitinib) represent a novel, oral class of tsDMARDs licensed for RA patients who are inadequate responders or intolerant to one or more DMARDs.^21^

Tofacitinib, the first in-class agent, is the one with the largest safety database with 6,194 patients who had been followed for 19,406 patient-years in its clinical development program (RCTs and their long-term extensions).^22^ Serious infections were observed in 8.5% of patients at a rate of 2.7/100 patient-years, which did not appear to increase during the long term follow-up.^22^ Factors associated with increased serious infection risk included baseline GC use, older age, presence of COPD or diabetes, higher HAQ score, higher BMI, severe lymphopenia (<500 cells/μL), male gender, line of therapy (3rd vs 2nd line), Asian origin of patients and higher tofacitinib dose.^22^ Up to now, based on the RCT and long-term extension data, the overall rate of serious infections of JAK inhibitors appears to be similar to that of bDMARDs, without major differences between the 3 approved JAK inhibitors.^17,18,23^ Post-marketing real life data for tofacitinib did not show any signals for an increased risk for serious infections [... for serious infections] neither.^24^

However, recently an interim analysis of a Phase 4, randomized, active-controlled, post authorization safety surveillance study, showed an increased risk of serious and fatal infections in RA patients > 50 years who had ≥1 cardiovascular risk factor (such as active smoking, hypertension, diabetes, family history of premature coronary artery disease, history of coronary disease and presence of extra-articular RA manifestations) and were treated with tofacitinib (5 or 10 mg twice a day) compared to those treated with a TNFi (etanercept or adalimumab).^25^ More specifically, the incidence rate of non-fatal serious infections was 3.51, 3.35 and 2.79/100 patients-years for tofacitinib 10 mg and 5 mg twice daily and TNFi respectively, whereas for fatal infections, the respective incidence rates were 0.22, 0.18 and 0.06/100 patient-years.^25^

The risk of serious infections (serious and non-serious) was further increased to patients over 65 years and based on these findings, a change in the drug’s SPC has been made by the EMA recently, stating that for patients >65 years old, tofacitinib should only be considered if no suitable alternative treatment is available.^25^

Although a full analysis of the study data has not been published and the findings apply so far to a specific RA subgroup (patients >50 years old with high-cardiovascular risk), caution is needed in RA patients with similar characteristics who are starting tofacitinib at the recommended dose (5 mg twice a day). So far, similar data for the other 2 approved JAK inhibitors are not available.

## POSTOPERATIVE PROSTHETIC JOINT INFECTIONS

The recent advances in the care of patients with RA have led to a decrease in the number of arthroplasties performed in these patients.^26,27^ Prosthetic joint infection (PJI) is a devastating and difficult-to-treat complication of joint arthroplasties with tremendous morbidity. Compared to osteoarthritis, RA patients have a similar risk for revision, but approximately 60% higher risk for PJI, without differences between bDMARD and non-bDMARD treated patients.^28^

In a recent RA cohort study covering the years 2006 through 2015, 30-day risk for serious infection and 1-year risk for PJI after total knee or hip arthroplasty were similar across different bDMARDs.^28^ In contrast, daily prednisone doses of >10 mg were associated with increased risk both for serious infections and PJI, underlying the need for preoperative GC tapering.

The American College of Rheumatology (ACR) and the American Association of Hip and Knee Surgeons recently published Guidelines on the appropriate perioperative timing of treatment discontinuation in rheumatic patients undergoing arthroplasties.^29^ Although practical and easy to use, all the included recommendations are conditional.

## OPPORTUNISTIC INFECTIONS

### Herpes zoster

Herpes zoster (HZ) is one of the most common viral infections in the aging population with a cumulative lifetime risk of 10–50%.^30^ Patients with RA have an almost 2-fold higher risk for HZ compared to the general population.^31,32^ The main risk factors include older age and the use of immunosuppressives.

A number of studies have shown that bDMARDs do not significantly increase the risk for HZ in RA patients compared to non-biologics^18,33^ without differences between the different biologic classes.^18,33 –35^

On the other hand, there is clearly a higher risk (∼2-fold) for HZ in RA patients treated with JAK inhibitors.^23,34^ In a recent pooled analysis of 44 studies with JAK inhibitors (26 with tofacitinib, 6 with baricitinib, 7 with upadacitinib, 5 with filgotinib) in different diseases such as RA, psoriasis, psoriatic arthritis, inflammatory bowel diseases and ankylosing spondylitis, the incidence of HZ was 2.11/100 patient-years among patients treated with JAK inhibitors compared to 1.23/100 patient-years in the comparator group.^36^ So far there is no clear difference regarding HZ risk between the 3 approved JAK inhibitors for RA (tofacitinib, baricitinib, upadacitinib).^23^

Tofacitinib, which was the first JAK inhibitor approved first by the FDA (2012) and later by the EMA (2017), has the most longitudinal data regarding HZ risk. In a recent analysis, approximately 11% of patients developed HZ (incidence rate: 3.9/100 patient-years).^22^ In the vast majority of cases (93–94%), HZ was classified as non-serious, involving only one dermatome.^37^ Co-administration of GCs, older age, and Asian origin were independent factors associated with HZ in this patient population.^22^

Almost all patients with HZ received antiviral therapy (90%) with a very low incidence of post-herpetic neuralgia (PHN, 7.4%) which is the most fearsome complication of HZ. Furthermore, ∼85% of patients continued tofacitinib therapy after their 1^st^ episode and among them ∼9% had a 2^nd^ HZ episode (96% non-serious).^37^

These data clearly show that increased vigilance for HZ is required for RA patients treated with JAK inhibitors. Overall, the clinical course of HZ in these patients appears to be benign with a low incidence of PHN if treated early with antiviral therapy. The universal use of the HZ vaccine in this population prior to the initiation of JAK inhibitors is expected to further decrease this risk (see vaccinations below).

### Tuberculosis

The introduction of TNFi in clinical practice led to a significant increase in cases of tuberculosis (TB) reactivation in patients with an undiagnosed, underlying latent TB infection (LTBI).^38^ Although TB reactivation was mainly associated with TNFi, TB cases have been reported at a lower rate with other bDMARDs^39,40^and tsDMARDs.^22^ However, the universal screening of patients prior to bDMARD initiation with the tuberculin skin test (TST) and/or the newer Interferon Gamma Release Assays (IGRAs), have led to a substantial decrease of newly diagnosed TB cases (∼80%).^39,41^

It is currently estimated that latent tuberculosis infection (LTBI) affects one quarter of the world population, with the respective prevalence in the Eastern Mediterranean basin of 16%.^42^ Recently, a similar LTBI prevalence (13–15%) has been found among 2491 Greek RA patients.^43^ In the general population, IGRAs are recommended over TST for LTBI screening, given their higher specificity and ease of use.^44^ However, for RA patients starting b- or ts-DMARDs, TST, IGRA or a dual screening strategy have been proposed (*Table 2*).^45–47^

**Table 2.** How to prevent serious infections in patients with rheumatoid arthritis treated with biologic or synthetic targeted DMARDs.

Baseline assessment of infectious risk - Age - Comorbidities (renal, lung, heart diseases, diabetes) - History of previous serious infections - Previous and current anti-rheumatic therapies (GCs, cs-, b- or ts-DMARDs) Pre-treatment screening for: - Tuberculosis (chest X-ray, TST and/or IGRA) - Hepatitis B virus infection (HBsAg, anti-HBc, anti-HBs) - Hepatitis C virus infection (anti-HCV) - IgG levels (prior to rituximab initiation) Vaccinations - Influenza virus Annually - Pneumococcus PCV13: Once PPSV23: < 65 years: 1 or 2 (5 years apart) doses > 65 --//--: 1 dose - Hepatitis B virus For high-risk, non-HBV exposed patients: 3 doses (at 0, 1 and 6 months) - Herpes zoster LZV: > 50 years: 1 dose (2-4 weeks prior to b-/ts-DMARD initiation) RZV: > 50 years: 2 doses (4-6 weeks apart) - Human papilloma virus Up to 26 years: 2 or 3 doses 26–45 years: 2 or 3 doses (shared clinical-decision making) During therapy - Minimize use of GCs (dose/duration) - Avoid contacts with definite or presumed transmissible infected persons - Good personal hygiene - Vigilant for signs of infections with appropriate and timely use of anti-microbial/-viral therapies

GC: Glucocorticoids, cs-conventional synthetic, b-biologic, ts- targeted synthetic DMARDs: biologic disease modifying anti-rheumatic drugs, TST: tuberculin skin test, IGRA: Interferon Gamma Release Assay, HBsAg: Hepatitis B surface antigen, anti-HBc: antibody against Hepatitis B core antigen, anti-HBs: antibody against HBsAg, anti-HCV: antibody against hepatitis C virus, PCV13: pneumococcal 13-valent conjugated vaccine, PPSV23: 23-valent pneumococcal polysaccharide vaccine, LZV: live zoster vaccine, RZV: recombinant zoster vaccine.

Despite appropriate baseline screening, cases of TB continue to be diagnosed worldwide during long term b- or ts-DMARD therapy.^39,40^ These cases are thought to be mainly due to TB re-exposure rather than reactivation. In the long-term extension studies of RCTs in RA patients treated with TNFi,^48^ non-TNFi^49^ bDMARDs or ts-DMARDs^22^, the incidence rate of TB ranged between 0.1 to 0.2/100 patient-years. In real life settings, the incidence rate depends mainly from the TB prevalence in the respective geographical areas. For example, in the UK, the incidence rate of TB in 2015 was 0.04/100 patient-years^39^ whereas in South Africa the respective rate was much higher at 1.2/100 patient-years.^40^

These observations raised a discussion of whether repeat TB re-screening is required for b- or ts-DMARD treated patients. So far, some scientific societies support repeat testing in high-risk populations only,^47,50,51^ whereas others do not offer specific guidance.^50^ Nevertheless, even in low TB prevalence countries, the majority of rheumatologists are still employing such retesting strategies.^52^ In a recent study in a low prevalence country such as Greece, we found that conversion of screening assays (TST, IGRA) during long-term bDMARD therapy was common and in most cases a transient event.^53^ Similarly, high conversion rates of IGRAs have been reported in health care workers during routine annual re-screenings and thus, the Centre for Disease Control (CDC) does not recommend them universally anymore, except for those at increased TB risk.^54^

Based on these findings, repeat TB testing is recommended only for high- risk patients or to those with suspicious clinical manifestations during long term b- or ts- DMARD therapy.^53^

All patients found positive with either TST or IGRA, should be treated for LTBI. Currently, suggested regimens are of shorter duration and include isoniazid (INH, 300 mg/day) for 6 months, rifampicin (RIF, 600 mg/day) for 4 months, or the combination of INH and RIF for 3 months.^44^ Regarding co-administration with JAK inhibitors, INH treatment has been shown to be well tolerated with low hepatotoxicity rates whereas drug interaction of tofacitinib with cytochrome P inducer RIF leads to a significant decrease to tofacitinib levels and should be avoided.

### Hepatitis B virus reactivation

Hepatitis B virus (HBV) remains the most common chronic viral infection worldwide.^55^ Its prevalence worldwide has been estimated at 3.6%,^55,56^ with a similar prevalence among RA patients, as it was shown recently in the International COMORA study (estimated prevalence: 3%).^57^

As it was the case with TB, early after the introduction of TNFi in clinical practice, a high rate of reactivation was noted in chronically infected HBV patients (HBsAg+) who had not received appropriate antiviral prophylaxis.^58,59^ In certain cases, HBV reactivation was severe leading to acute hepatitis, liver failure and even death (especially among cirrhotic patients).^58,59^ Later on, the prophylactic administration of appropriate oral antiviral therapy diminished the rate of HBV reactivation during b-DMARD therapy.^60^ Cases of HBV reactivation has been reported with all immunosuppressive agents used in the treatment of RA, including GCs, b- and ts-DMARDs.^56^

Currently, all RA patients starting DMARD therapy should be screened for HBV infection with HBsAg, anti-HBc and anti-HBs antibodies (*Table 2*).^47,56^ Such screening offers the opportunity of identifying susceptible patients, while vaccination should be offered in HBV negative patients (HBsAg-, anti-HBc-, anti-HBs-) who are at high risk for HBV exposure.^56^

Patients who are HBsAg+ (chronic infection) should receive appropriate chronic antiviral prophylaxis with the newer generation of oral antivirals (entecavir, tenofovir disoproxil fumarate or alafenamide).^56^ For those with past or resolved HBV infection (HBsAg-, anti-HBc+, anti-HBs±), close monitoring of HBsAg, HBV DNA and ALT levels is recommended (especially for patients who are treated with B cell depleting agents)^56^ with antiviral prophylaxis initiation in case of HBV reactivation (HBsAg or HBV DNA+).

### Vaccinations

Vaccinations remain the most efficient and cost-effective measure for prevention of different bacterial or viral infections and are currently recommended also for patients with different rheumatic diseases.^61^ In *Table 2*, the most commonly needed vaccinations in adult patients with RA are shown. Despite their proven efficacy and safety, vaccination uptake in rheumatic patients is low undermining the prevention of several serious infections.^61^

### Pneumococcal – Influenza vaccinations

Current recommendations include annual influenza vaccination and a combined serial vaccination algorithm that includes both the pneumococcal 13-valent conjugated (PCV13: once in a lifetime) and the 23-valent polysaccharide (PPSV23: 1 or 2 doses before the age of 65 and 1 dose after 65 years) vaccines (*Table 2*).

In our recent multi-centre RA study^43^, the rate of pneumococcal and last year’s influenza vaccination was 36% and 32%, respectively indicating a significant deficit of preventive strategies and abundant space for interventions, as also shown in other recent studies.^62^ Any concerns for disease flares after vaccination are unsubstantiated and should not prevent rheumatologists from vaccine prescription, since several studies have not showed such correlation.^63,64^

Data regarding the potential effect of different anti-rheumatic therapies (GCs, cs-, b or ts-DMARDs) in vaccination efficacy have provided conflicting results (reviewed in ref. ^64^). Despite the negative impact of some DMARDs on immunogenicity,^64^ the net benefit of vaccinations in rheumatic patients is beyond the shadow of a doubt.

In a recent analysis, Adami et al. showed similar reductions in influenza rates between healthy individuals and RA patients on TNFi (59% and 69%, respectively) after influenza vaccination.^65^ Interestingly, the number needed to vaccinate (NNV) to prevent one case of influenza was significantly lower in the RA compared to the healthy control group (10 vs. 71).^65^

### Human papilloma virus vaccination

Human papilloma virus (HPV) vaccination has been one of the most successful preventive interventions in history, completely transforming the landscape of one of the most common cancers, cervical cancer. Although the number of young patients with RA is relatively small, rheumatologists should keep in mind that RA patients carry a higher risk for low- and high-grade squamous intraepithelial lesions (SIL)^66^ whereas in those treated with bDMARDs the risk for high-grade SIL and invasive cervical cancer may be increased by 2-fold.^67^

According to the most recent US Guidelines for the general population, all young patients with RA up to 26 years old should be vaccinated with 2- or 3-dose series depending on age at initial vaccination or condition whereas for those between the ages of 27–45 years a shared clinical decision should be made (*Table 2*).^68^

### Herpes zoster vaccination

The introduction of the live attenuated zoster vaccine (LZV) in clinical practice was a significant milestone, as it was the first vaccine to prevent the reactivation of a latent infection. LZV has been shown to decrease HZ incidence by 50% and PHN (its most frequent and debilitating complication) by 67% with an estimated duration of protection of 5–7 years.^61^ Although LZV administration was reported to be safe in patients on bDMARDs (mainly TNFi)^69^, current recommendations propose to administer 1 dose of LZV in patients older than 50 years, 2–4 weeks prior to initiation of b- or ts-DMARDs (table 2).^47,61^

A new type of recombinant subunit zoster vaccine (RZV) which is highly immunogenic (due to an adjuvant activating toll-like receptors) has been recently launched, showing significantly higher protection rates against HZ and PHN.^68^ The vaccine is currently recommended for adults older than 50 years, including immunosuppressed patients and is administrated in 2 doses (2 to 6 months apart, *Table 2*).^61,68^ Recent results in various immunosup-pressed populations^70–72^ and patients with IBD73 have shown a favourable vaccine efficacy and safety profile.

## CONCLUSIONS

In the emerging new era of targeted synthetic therapies for RA, serious infections continue to remain the most important complication of chronic therapy. Appropriate baseline screening (HBV, TB), pre- and on-treatment vaccinations for certain pathogens (pneumococcal, herpes zoster, flu vaccines), aggressive therapy for RA in order to achieve adequate control of disease activity as well as continuous vigilance for early signs of infections are needed in all RA patients. Special attention is required in older RA patients (>65 years) with co-morbidities who are in general more prone to infectious complications regardless of their treatment.
